# Non‐Cultured Melanocyte‐Keratinocyte Transplantation Combined With NB‐UVB Therapy for Facial Depigmentation Induced by Skin Exfoliator: A Case Report

**DOI:** 10.1002/ccr3.70840

**Published:** 2025-09-14

**Authors:** Sona Zare, Alireza Jafarzadeh, Maryam Nouri, Solmaz Zare, Mohammad Ali Nilforoushzadeh

**Affiliations:** ^1^ Skin and Stem Cell Research Center Tehran University of Medical Sciences Tehran Iran; ^2^ Pars Fundamental Bio Structure Company Tehran Iran; ^3^ Stem Cell and Regenerative Medicine Institute Sharif University of Technology Tehran Iran; ^4^ Department of Mechanical Engineering Sharif University of Technology Tehran Iran; ^5^ Skin Repair Research Center Jordan Dermatology and Hair Transplantation Center Tehran Iran; ^6^ Department of Dermatology, Rasool Akram Medical Complex Clinical Research Development Center (RCRDC) School of Medicine, Iran University of Medical Sciences Tehran Iran; ^7^ Laser Application in Medical Sciences Research Center Shahid Beheshti University of Medical Sciences Tehran Iran

**Keywords:** cell therapy, keratinocyte, melanocytes, non‐cultured, transplantation

## Abstract

Non‐cultured melanocyte‐keratinocyte transplantation combined with narrowband ultraviolet B (NB‐UVB) therapy is a promising and well‐tolerated treatment for facial hypo‐pigmentation following scarring. This approach has shown significant improvement in patients who have not responded to other therapies.

## 1 Introduction

Scar formation in the skin is a natural response that aligns with the healing process of the skin layers following a traumatic incident. This complex process involves inflammation, proliferation, and remodeling phases, ultimately leading to the formation of fibrotic tissue. A characteristic feature of facial scarring is typically the degeneration of melanocytes, resulting in pigmentary changes such as hypopigmentation or hyperpigmentation [1, 2]. These pigmentary alterations are often more pronounced in facial scars due to the region's high vascularity and constant exposure to environmental factors such as ultraviolet (UV) radiation [3].

Facial scarring is not only an aesthetic concern but can also have significant psychosocial implications. It may affect self‐confidence, social interactions, and overall mental well‐being [3]. Studies have shown that individuals with visible facial scars are more prone to emotional distress, emphasizing the importance of effective treatment strategies [4].

Various treatment approaches have been explored to improve the appearance of facial scars and restore pigmentation. These include topical and systemic medications, phototherapy sessions, laser therapy, cell therapy, and surgical interventions [5]. While these options can provide varying degrees of improvement, outcomes are often limited, particularly in cases of deep or long‐standing scars [4].

Regenerative medicine offers innovative treatments for refractory facial scars, with the Melanocyte‐Keratinocyte Transplantation Procedure (MKTP) gaining attention for its ability to restore pigmentation. By transplanting a melanocyte‐keratinocyte cell suspension, MKTP improves color matching and aesthetic outcomes compared to traditional grafting methods [6].

However, the success of MKTP is influenced by several factors, including the anatomical characteristics and accessibility of the scar, the formulation of the cell suspension, the use of adjunctive therapies such as phototherapy, and the specific transplantation technique employed [7]. Understanding these variables is essential for optimizing treatment outcomes and ensuring patient satisfaction.

In this case report, we present the successful use of non‐cultured melanocyte‐keratinocyte transplantation combined with Narrow‐Band Ultraviolet B (NB‐UVB) therapy for treating facial depigmentation induced by a skin exfoliator in a 30‐year‐old woman.

## 2 Case History/Examination

**FIGURE 1:**
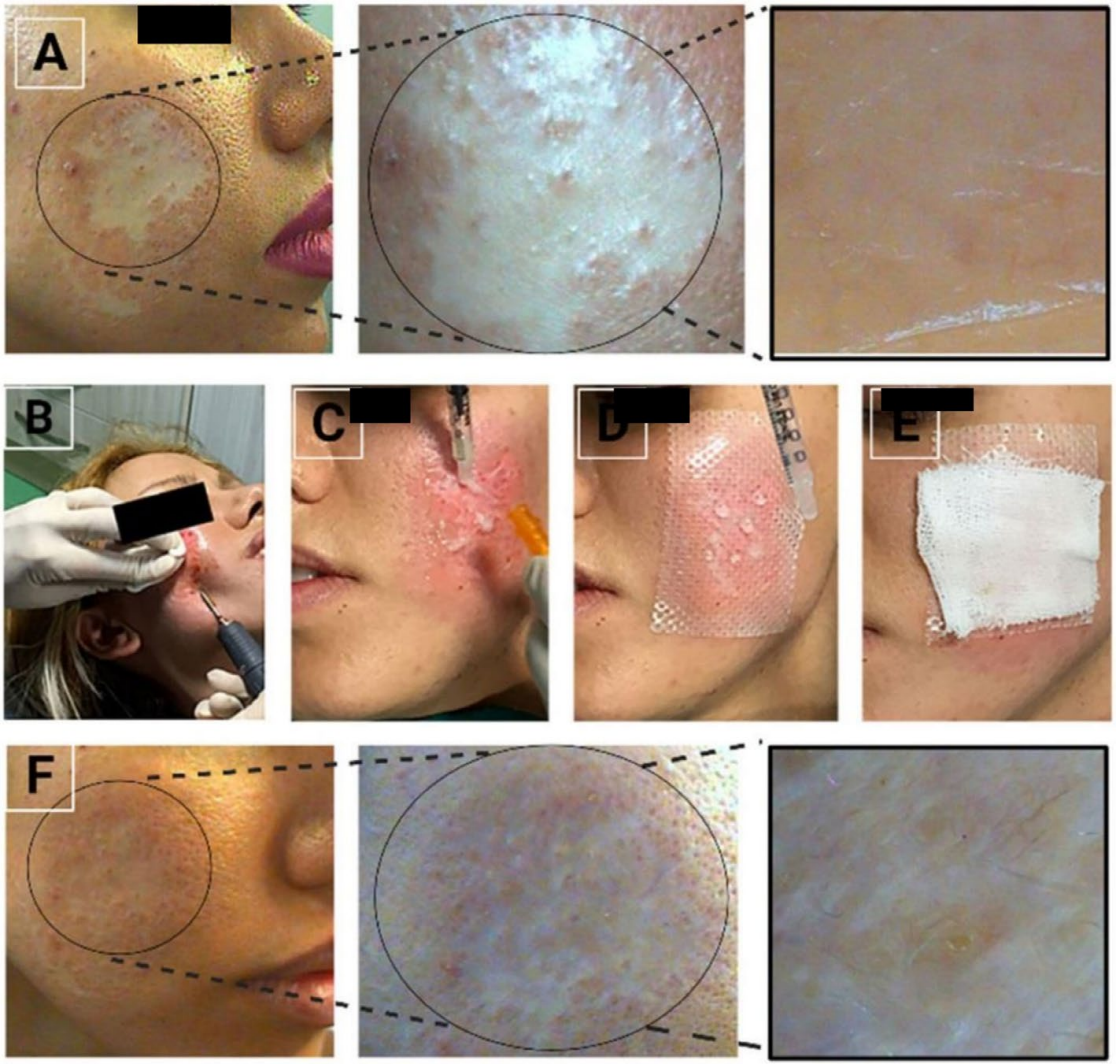
The patient had a history of facial de‐pigmentation following exposure to a facial exfoliator (A). The patient subsequently underwent microdermabrasion (B). A cell suspension was applied to the target site (C), which was then covered with appropriate dressings (D, E). The re‐pigmentation following transplantation is illustrated at different magnifications (F).

The patient is a 30‐year‐old woman with a history of depression and prior use of antidepressant medications. She recently applied salicylic acid as a facial exfoliator, which resulted in hypopigmentation and skin discoloration in the treated areas (Figure 1A). Before her visit, she had used mometasone ointment and tacrolimus ointment in an attempt to manage the discoloration. After an interval of 2 months following the onset of depigmentation, the patient was considered a candidate for non‐cultured melanocyte‐keratinocyte transplantation.

## 3 Methods

The application of the cell suspension was consistent with the patent application. Following local anesthesia, a 2 × 5 cm sample with a depth of 1 mm was collected from the patient's gluteal region. The skin sample was placed in alcohol and phosphate‐buffered saline (PBS) without antibiotics. It was then cut into segments and treated with Trypsin LE Select enzyme (TrypLETM solution) for 45 min. The solution was subsequently diluted with PBS (1:5) to deactivate the enzyme. Afterward, the suspension was passed through a 100 μm mesh filter and centrifuged for 10 min at 1500 rpm. Following centrifugation, 2 cc of PBS was added to the cell pellet.

The target region was approached for abrasion using a microdermabrasion unit (Tebmax x18, Nozhan Co, Iran), followed by washing with physiological fluid (Figure 1B). The cell suspension was then evenly applied over the injured site (Figure 1C). A Mepitel wound dressing (Mepitel, Mölnlycke, Sweden) was placed over the area and covered with a Tegaderm transparent dressing (Figure 1D,E). The patient was discharged after a few hours, and the dressings were removed after 7 days. Starting from the 14th day post‐transplantation, the patient received 30 sessions of NB‐UVB treatment.

## 4 Conclusion and Results

After a three‐month period, significant improvement in facial hypopigmentation was observed based on image analysis. Additionally, the subjective assessment indicated that the results were satisfactory for the participant in this study (Figure 1F).

## 5 Discussion

MKTP offers a promising treatment for patients unresponsive to other therapies by transferring melanocytes to hypo‐pigmented areas [8]. In the current case, MKTP was successfully used to treat localized facial hypopigmentation, demonstrating favorable outcomes consistent with those reported in previous literature.

For instance, Shahbazi concluded that the healing rate among patients with focal vitiligo was significantly higher than that of those with diffuse vitiligo [6], a finding echoed in this case, where localized lesions responded well post‐procedure. This aligns with reports stating that non‐cultured melanocyte‐keratinocyte transplantation can result in up to 90% repigmentation in 65% of patients with focal vitiligo [9].

Similarly, Nuntawisuttiwong et al. [7] supported the idea that focal vitiligo is more responsive than generalized vitiligo, further validating the favorable outcome observed in our patient. Wang et al. [9] also demonstrated that three sessions of melanocyte transplantation with punch grafting led to 90% improvement in pretibial hypopigmentation, showing how repeated procedures could amplify success—relevant to the current case, which showed progressive repigmentation within a few months.

Tawfik's comparative study of two MKTP methods revealed that higher cell density (3000 cells/mm^2^) yielded better outcomes than lower density (1000 cells/mm^2^), especially regarding VASI score and color match [10]. Our case utilized a cell‐rich suspension and achieved comparable success, further reinforcing the importance of optimizing cell concentration. Although NB‐UVB adjuvant therapy provided only modest improvement in Tawfik's study, it complemented outcomes in our case by enhancing pigment uniformity post‐operatively.

In a prospective open‐label study involving 28 patients, autologous MKTP showed a range of repigmentation outcomes—from excellent (17%) to poor (41%) [11]. Our patient achieved over 75% repigmentation, placing them in the “good” to “excellent” range and highlighting MKTP's consistent efficacy in focal lesions.

MKTP also proves effective in post‐burn leucoderma cases, where its donor‐to‐recipient ratio (1:10) and minimal scarring make it suitable for larger areas [12]. Our case, although facial and non‐traumatic, mirrors these benefits in terms of aesthetic improvement and minimal invasiveness.

Notably, repigmentation in most studies—including ours—typically occurs 2 to 4 months after multiple sessions [13, 14, 15], underscoring the need for patient patience and adherence. Patients with focal vitiligo or traumatic hypopigmentation—like the one treated here—benefit most from MKTP, especially when prior treatments (e.g., steroids or laser therapy) fail.

Compared to traditional treatments like corticosteroids, calcineurin inhibitors, and laser therapies—which may show inconsistent results in chronic or deep‐seated lesions—MKTP offers minimal invasiveness, autologous cell compatibility, and shorter recovery time [5, 16]. Our patient resumed daily activities within days, reporting no scarring or significant downtime.

Mechanistically, MKTP involves harvesting melanocytes and keratinocytes from a pigmented donor site and applying them to the hypo‐pigmented region. Adjunctive NB‐UVB therapy, as used in our patient, enhances melanocyte proliferation and survival while reducing autoimmune responses, leading to uniform repigmentation and reduced relapse rates [5, 17].

Despite these advantages, MKTP carries some limitations, such as patient variability and fibrosis, which can affect outcomes. Although not observed in our case, these risks reinforce the need for long‐term follow‐up to assess pigment retention and detect any delayed complications. Future larger‐scale studies are warranted to validate the findings and further explore the procedure's potential in broader clinical use.

## Author Contributions

**Sona Zare:** conceptualization, investigation, visualization. **Alireza Jafarzadeh:** methodology, writing – original draft, writing – review and editing. **Maryam Nouri:** data curation, formal analysis, software. **Solmaz Zare:** data curation, validation, writing – original draft. **Mohammad Ali Nilforoushzadeh:** conceptualization, project administration, supervision, visualization.

## Disclosure

Transparency declaration: Authors declare that the manuscript is honest, accurate, and transparent. No important aspect of the study is omitted.

## Ethics Statement

The researchers were committed and adhered to the principles of the Helsinki Convention and the Ethics Committee of the Iran University of Medical Sciences in all stages. The procedure was approved by the Ethics Committee of Tehran University of Medical Sciences with the ethical code IR.TUMS.FMD.REC.1400.0623, dated 2021‐05‐02.

## Consent

After providing the necessary explanations, written informed consent was obtained from the patient regarding the submission of their clinical condition to medical journals. Additionally, the patient has been assured that their name and personal details will be kept confidential by the authors.

## Conflicts of Interest

The authors declare no conflicts of interest.

## Data Availability

The data that support the findings of this study are available on request from the corresponding author. The data are not publicly available due to privacy or ethical restrictions.
